# Exploring the feasibility of pupillometry training and perceptions of potential use for intracranial pressure monitoring in Uganda: A mixed methods study

**DOI:** 10.1371/journal.pone.0298619

**Published:** 2024-05-15

**Authors:** Zoey Petitt, Yesel Trillo Ordonez, Chibueze Agwu, Maura Ott, Muhammad Shakir, Alexandria Ayala Mullikin, Jenna Davis, Adham M. Khalafallah, Alan Tang, Chidyaonga Shalita, Joseph Mary Ssembatya, Di D. Deng, Jennifer Headley, Oscar Obiga, Michael M. Haglund, Anthony T. Fuller

**Affiliations:** 1 Duke University Division of Global Neurosurgery and Neurology, Durham, NC, United States of America; 2 Duke University School of Medicine, Durham, NC, United States of America; 3 Duke University Global Health Institute, Durham, NC, United States of America; 4 Pritzker School of Medicine, The University of Chicago Medical Center, Chicago, IL, United States of America; 5 Aga Khan University Hospital, Karachi, Pakistan; 6 University of Miami Miller School of Medicine, Miami, FL, United States of America; 7 Department of Neurosurgery, University of Miami, Miami, FL, United States of America; 8 Division of Neurosurgery, Mbarara Regional Referral Hospital, Mbarara, Uganda; 9 Department of Neurosurgery, Mulago National Referral Hospital, Kampala, Uganda; 10 Department of Neurosurgery, Duke University Medical Center, Durham, NC, United States of America; North Memorial: North Memorial Health, UNITED STATES

## Abstract

**Introduction:**

Traumatic brain injury (TBI) accounts for the majority of Uganda’s neurosurgical disease burden; however, invasive intracranial pressure (ICP) monitoring is infrequently used. Noninvasive monitoring could change the care of patients in such a setting through quick detection of elevated ICP.

**Purpose:**

Given the novelty of pupillometry in Uganda, this mixed methods study assessed the feasibility of pupillometry for noninvasive ICP monitoring for patients with TBI.

**Methods:**

Twenty-two healthcare workers in Kampala, Uganda received education on pupillometry, practiced using the device on healthy volunteers, and completed interviews discussing pupillometry and its implementation. Interviews were assessed with qualitative analysis, while quantitative analysis evaluated learning time, measurement time, and accuracy of measurements by participants compared to a trainer’s measurements.

**Results:**

Most participants (79%) reported a positive perception of pupillometry. Participants described the value of pupillometry in the care of patients during examination, monitoring, and intervention delivery. Commonly discussed concerns included pupillometry’s cost, understanding, and maintenance needs. Perceived implementation challenges included device availability and contraindications for use. Participants suggested offering continued education and engaging hospital leadership as implementation strategies. During training, the average learning time was 13.5 minutes (IQR 3.5), and the measurement time was 50.6 seconds (IQR 11.8). Paired t-tests to evaluate accuracy showed no statistically significant difference in comparison measurements.

**Conclusion:**

Pupillometry was considered acceptable for noninvasive ICP monitoring of patients with TBI, and pupillometer use was shown to be feasible during training. However, key concerns would need to be addressed during implementation to aid device utilization.

## Introduction

Traumatic brain injury (TBI) is a leading cause of morbidity and mortality worldwide [1, 2]. It has been estimated that up to 74 million people will experience a new TBI annually, [2] with incidence and prevalence increasing globally [3]. Compared to high-income countries (HICs), low-and middle-income countries (LMICs) have three times the number of cases of TBI proportionally, [2] more risk factors for TBI, and less adequately prepared health systems for TBI management [1]. In Uganda, a low-income country in East Africa, TBI mortality was estimated to be 9.6%, with mortality rates of 4.7% for patients with mild and moderate TBI and 55% for patients with severe TBI in 2016. At Mulago National Referral Hospital (MNRH) in Kampala, Uganda, factors that are known to impact mortality, [4, 5] such as delays in seeking and receiving care, have been observed for patients with TBI [6].

Elevated intracranial pressure (ICP) is a common mechanism for adverse outcomes from TBI; thus, monitoring ICP is important for TBI management and has been shown to improve outcomes [7]. In HICs, invasive monitoring is the standard of care for ICP monitoring [8]. Though invasive ICP monitoring is more accurate and can provide treatment, these methods increase the risk of infection, hemorrhage, and neurological deficit [9, 10]. Noninvasive ICP monitoring methods include fluid dynamic methods like magnetic resonance imaging and transcranial doppler ultrasound, ophthalmologic methods like pupillometry and optic nerve ultrasound, otic methods, and electrophysiological methods [9, 10]. In limited-resource settings, such as Uganda, invasive ICP monitoring techniques are often inadequate or unavailable due to the lack of infrastructure required for safe, routine use [11]. Currently, no noninvasive ICP monitoring devices are used clinically in Uganda, and more information is needed on existing monitoring methods.

Pupillometry is portable, relatively low-cost compared to invasive monitoring, and a quick tool that measures pupillary response to light. The NPi®-200 Pupillometer, manufactured by Neuroptics®, is a handheld pupillometer that requires disposable SmartGuards® and completes a measurement of each eye in 3–4 seconds [12]. It stimulates the pupil light reflex with a fixed intensity light and uses infrared to record and quantify the pupil response [13, 14]. The device reports multiple aspects of the pupil response to light, including the pupil size and neurological pupil index^TM^ (NPi®), [13, 14] a standardized metric that classifies the pupil response as normal or abnormal [15]. Compared to the manual pupil exam, pupillometry has better precision, reproducibility, and interobserver and intraobserver reliability [14, 16]. Pupillometry can detect subtle changes in pupil measures not seen on manual exam, [14] before they are detected on manual exam, [14] or before a peak in ICP is seen [17].

Due to the impact of elevated ICP on the pupil light response, [17–19] pupillometry has also been evaluated as a form of noninvasive ICP monitoring. In previous studies in HICs, abnormal NPi® was associated with elevated ICP, as confirmed by invasive ICP monitoring in patients with and without TBI [20–24]. In patients with severe TBI, abnormal NPi® values were associated with increased ICP that returned to normal after treatment, and a higher burden of abnormal NPi® values was associated with worse outcomes [18]. Despite this evidence of an association between elevated ICP and abnormal NPi® values, pupillometry has not yet been implemented clinically for noninvasive ICP monitoring. It is typically only used as an adjunct for the neurological exam in HIC settings, with no published reports of its sole use for ICP monitoring in any setting.

In Uganda, there are no noninvasive monitoring devices currently being used for ICP monitoring. Noninvasive ICP monitoring has the potential to transform patient care in limited-resource settings by providing a safe, low-cost form of ICP monitoring to be used alongside clinical monitoring. This could change TBI care by decreasing the time to diagnose and treat elevated ICP, given that pupillometry can detect changes more quickly than the clinical exam. Because pupillometry has not been previously utilized in a clinical setting or studied in Uganda, this study is necessary to evaluate the feasibility of pupillometry before implementation. This study examines the feasibility of pupillometry for noninvasive ICP monitoring at MNRH and aims to assess the perception of pupillometry, fidelity of pupillometry measurements after training, and barriers and facilitating factors for clinical implementation.

## Methods

To assess the feasibility of pupillometry for patients with TBI, a mixed methods study was performed to evaluate key components of the feasibility of the introduction of new technology, including perception, implementation, and fidelity. In this study, healthcare providers at MNRH received education on pupillometry and completed interviews on their perspectives. Initial interviews were followed by a training session where fidelity measurements were taken and a second interview on the perception of pupillometry implementation. The education session, individual interviews, and pupillometry training session were all conducted by the same researcher (ZP) in order to develop a relationship with participants over time and create standardization in the responses and results obtained.

### Setting

The study was completed at MNRH, a public hospital located in Kampala, Uganda, with an established neurosurgery department. At MNRH, this study worked with the Neurosurgery department and the Accident and Emergency ward, where care is provided for patients with mild to moderate TBI. These locations were the focus of this study due to the study’s goal of assessing the utility of pupillometry for detecting a new elevation in ICP. The Neurosurgery department and the Accident and Emergency ward were best suited for this study, as patients more commonly present without elevated ICP and require monitoring. Patients with severe TBI and known elevated ICP are often treated in the intensive care unit (ICU). Therefore, the ICU was not included in this study. Additionally, it was thought that differences in the clinical condition, care needs, and co-existing injuries in patients in the ICU may lead to differences in the use of pupillometry in this patient population. This study involved most neurosurgeons and neurosurgical residents who work on these wards, so many of these providers could not contribute as co-authors to reduce biases such as confirmation bias, self-serving bias, and sampling bias. Data collection activities occurred from July 20th, 2022, through August 15th, 2022.

### Participants

Neurosurgeons, neurosurgery and general surgery residents, and nurses were included in the study if they worked directly with patients with TBI in their role. Laboratory staff were included if they worked with samples from patients with TBI in their role. Although laboratory staff do not provide patient care, they were included as participants to enhance provider diversity in the study, thus increasing the scope of perspectives and feasibility data collected. Participants were excluded if they did not work full-time or had worked in their current position for less than four weeks. Purposive sampling was used for sample selection. All eligible providers were invited to participate during the Neurosurgery department meeting or were nominated by supervisors in July and August, 2022.

### Sample size estimation

For the qualitative component of the study, it was expected that 12–16 interviews would allow the study to reach at least 90% saturation [25–27]. In the fidelity portion of the study, estimates of sample size were obtained based on expected values for accuracy of pupillometry, which was previously found to be 99% [28]. The target sample size was calculated to be 16 measurements. Thus, this study aimed to recruit at least 20 providers to allow for a dropout rate of up to 20% and ensure there were at least 16 participants to complete the study.

### Education session

The education session was delivered by one researcher (ZP) as a 30-minute presentation covering topics including TBI burden of disease, the pathophysiology of elevated ICP, ICP monitoring, pupillometry, and the association between pupillometry measurements and elevated ICP. Participants were encouraged to ask clarifying questions as needed.

### Individual interviews

After completing the education session, all participants completed an individual pre-training interview to discuss their perspectives on pupillometry. A second post-training interview was completed to discuss perceived barriers and facilitating factors for using pupillometry clinically. The laboratory staff were excluded from the post-training interview because they did not provide direct care to patients in their role.

Both interviews followed semi-structured interview guides with open-ended and probing questions used as needed (S1 Interview Guides in S1 File). Participants were instructed in the interviews to provide both positive and negative perceptions. One researcher (ZP) conducted interviews at varying locations in MNRH and at a nearby private office according to each participant’s availability. The average time to complete the interviews was 37 minutes (pre-training) and 30 minutes (post-training). Interviews were conducted in English, recorded with audio recording devices, and then transcribed by a team of researchers (ZP, MS, MO). All transcripts were reviewed for accuracy and de-identified before data analysis.

### Pupillometry training session

To improve participant understanding of pupillometry and assess the fidelity of pupillometry measurements, participants engaged in a 2-hour training session after the completion of their first interview and before their second interview. Each training session included 2–5 participants. The training session included instruction on device use, demonstrations from a trainer (ZP), and hands-on practice. The study participants and the trainer served as healthy volunteers for the practice portion. Each participant obtained 16 paired pupillometry measurements on the healthy volunteers during the practice portion. Then, the trainer completed a comparison measurement on the same volunteer in the same location using the same device. The NPi® and maximum pupil size for the comparison measurements were recorded.

### Measures

Fidelity of pupillometry was assessed with learning time, time to obtain a measurement, and accuracy of measurements for NPi® and pupil size. The learning time was recorded as the time it took a participant to complete 16 bilateral measurements during training. The time to obtain a measurement was calculated by dividing the learning time by the number of practice measurements. The accuracy of measurement was assessed by comparing the measurement obtained by the participant with a measurement obtained immediately after by the trainer (ZP). Accuracy was evaluated using two types of cut-offs. For the clinically relevant cut-off, a measurement was considered accurate if the difference between the participant’s measurement and the trainer’s measurement was within 0.5 for consistency with previous literature [29]. For the device error cut-off, a measurement was considered accurate if the difference between the participant’s and the trainer’s measurements was within the potential device error of 0.03mm [15] for pupil size. There was no known device error for NPi®. The proportion of accurate measurements for NPi® and pupil size for each eye was calculated using these cut-offs.

### Data analysis

Qualitative data were analyzed using inductive coding and the framework analysis method [30]. To develop the codebook, eight transcripts were selected for initial review. After inductive coding of the first eight transcripts, an inclusive codebook was developed and used to code all transcripts. If new themes were identified after initial codebook development that were relevant to the research aims, these were added to the codebook during the coding process. Coding was completed by a team of five researchers (ZP, MS, MO, CA, YT) using Microsoft Excel [31]. One researcher coded each transcript, and then all researchers met to discuss the application of codes and reach agreement for each transcript.

For further analysis, codes and text segments were aggregated into additional Excel sheets and separated by parent code, primary child code, and secondary child code. Code frequency percentages were calculated as the number of participants that discussed the code at least once divided by the total number of participants. There were a few topics relating to overall perception that aligned with a specific prompt in the post-training interview, so the code frequencies for these codes were calculated out of the number of participants who completed the post-training interview. Participant knowledge was also assessed by determining if participants discussed key topics related to pupillometry during their interviews. Descriptive statistics were utilized to analyze participants’ demographic information.

For the fidelity measurements, paired t-tests evaluated if there was a statistically significant difference between the NPi® and pupil size comparison measurements. The mean and IQR were calculated for learning time and time to obtain a measurement.

### Ethical statement

The study was reviewed and approved by the authors’ Institutional Review Board and Mulago National Referral Hospital Research and Ethics Committee. All participants provided written informed consent and received compensation for their time.

### Inclusivity in global research

Additional information regarding the ethical, cultural, and scientific considerations specific to inclusivity in global research is included in the Supporting Information (S2 Checklist in S1 File).

## Results

### Sample description

**Fig 1:**
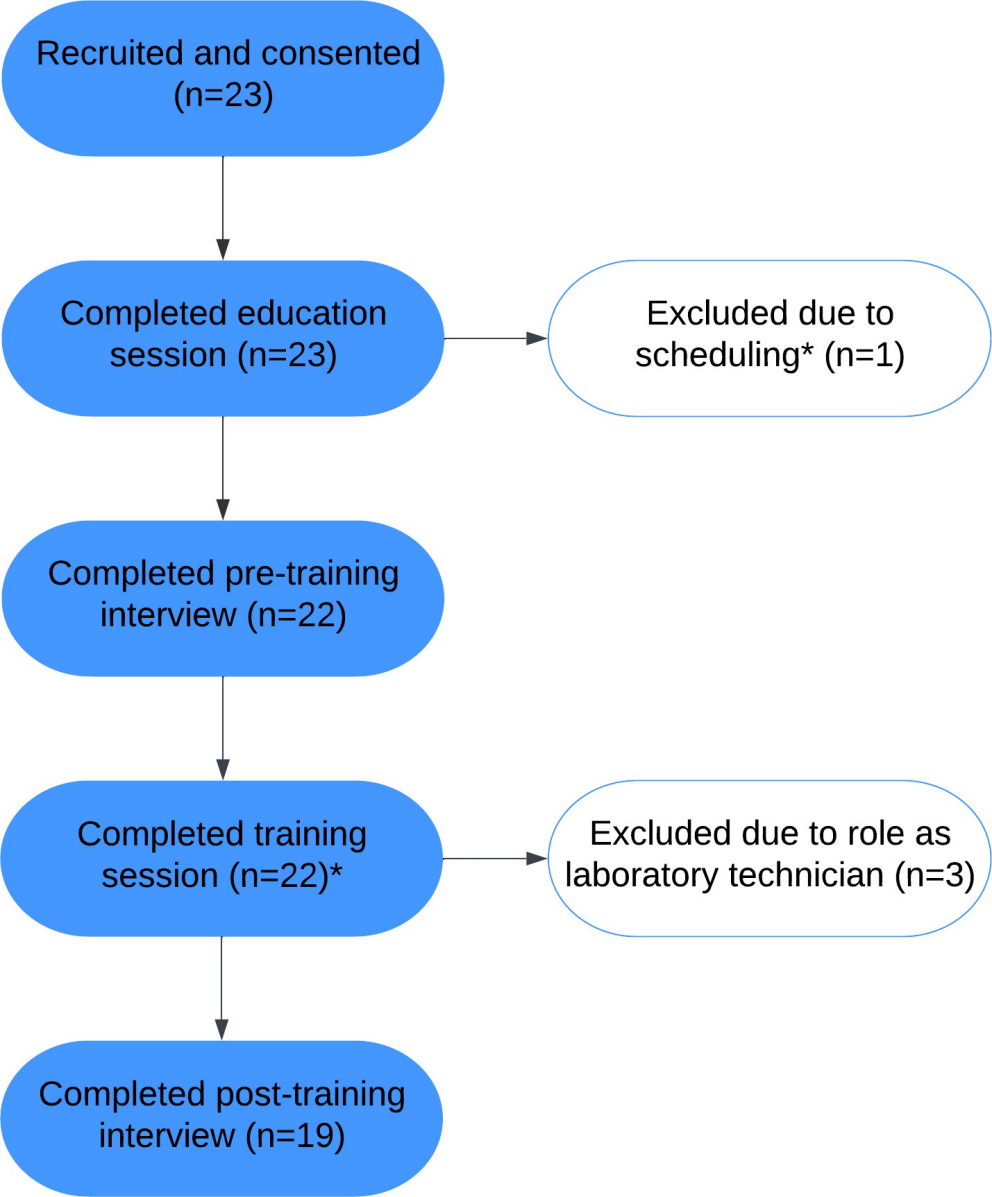
Number of participants who completed each component of the study. *One participant was excluded after the education session due to difficulty scheduling remaining study components. During the training session, one participant did not complete the number of measurements specified in the study protocol, so their fidelity measurement results were excluded from analysis.

Overall, 23 providers were recruited to participate (Fig 1). One neurosurgeon did not complete the study due to scheduling conflicts and was therefore excluded. Twenty-three participants completed the education session, and 22 completed both the pre-training interview and the pupillometry training session. Of the participants who completed the training session, one resident completed a different number of practice measurements than specified in the study protocol, so their fidelity measurement data were excluded. The three laboratory providers were excluded from the post-training interview, so 19 participants were eligible for this interview and completed it.

The 22 providers who completed the study included three neurosurgeons, seven residents, nine nurses, and three laboratory technicians (Table 1). Of the neurosurgeons, two worked in the emergency ward, and one worked in the neurosurgery ward. Three residents were general surgery residents completing their neurosurgery rotation, while four were neurosurgery residents. Four nurses worked in the neurosurgery ward, and five worked in the emergency ward.

**Table 1:** Demographic information of all study participants and those included in the post-training interview.

Category		Overall Value (%)	Participants who Completed Post-Training Interview Value (%)
Demographics	Total participants	22 (100)	19 (100)
	Female	9 (41)	9 (47)
	Male	13 (59)	10 (53)
	Age (years), average (IQR)	38.7 (10)	36.9 (7.5)
Role	Nurse	9 (41)	9 (47)
	Resident	7 (32)	7 (37)
	Neurosurgeon	3 (14)	3 (16)
	Laboratory technician	3 (14)	0 (0)
Primary Department	Neurosurgery	9 (41)	9 (47)
	Emergency	7 (32)	7 (37)
	General Surgery	3 (14)	3 (16)
	Laboratory	3 (14)	0 (0)
Experience	Years in current role, average (IQR)	10.3 (14.7)	8.6 (6.75)
	Years in current department, average (IQR)	5.2 (4.5)	4.6 (4.3)
	Interacts directly with patients	20 (91)	19 (100)

### Participant knowledge

After the education session, most providers (n = 21, 95%) could describe the importance of ICP monitoring for the care of patients with TBI, and 20 providers (91%) discussed how elevated ICP is associated with poor outcomes for patients. Fewer participants (n = 14, 64%) accurately defined the pupil light response; however, most (n = 17, 77%) could explain the relationship between elevated ICP and the pupil light response. When discussing pupillometry, most participants were able to recall the pupillometer (n = 16, 73%) and describe the association between elevated ICP and pupillometry (n = 18, 82%) (S3 Table in S1 File).

### Overview of interviews

In both qualitative interviews, overarching themes were identified in perception (S4 Fig in S1 File) and implementation of pupillometry (S5 Fig in S1 File). Perception themes were related to provider perceptions of noninvasive ICP monitoring for use as a clinical decision-support tool in the care of patients with TBI at MNRH. Implementation themes were related to the perception of clinical implementation of pupillometry for ICP monitoring. All themes and frequencies along with quotations from participants are reported in S6-S9 Tables in S1 File.

### Perception of pupillometry

**Fig 2:**
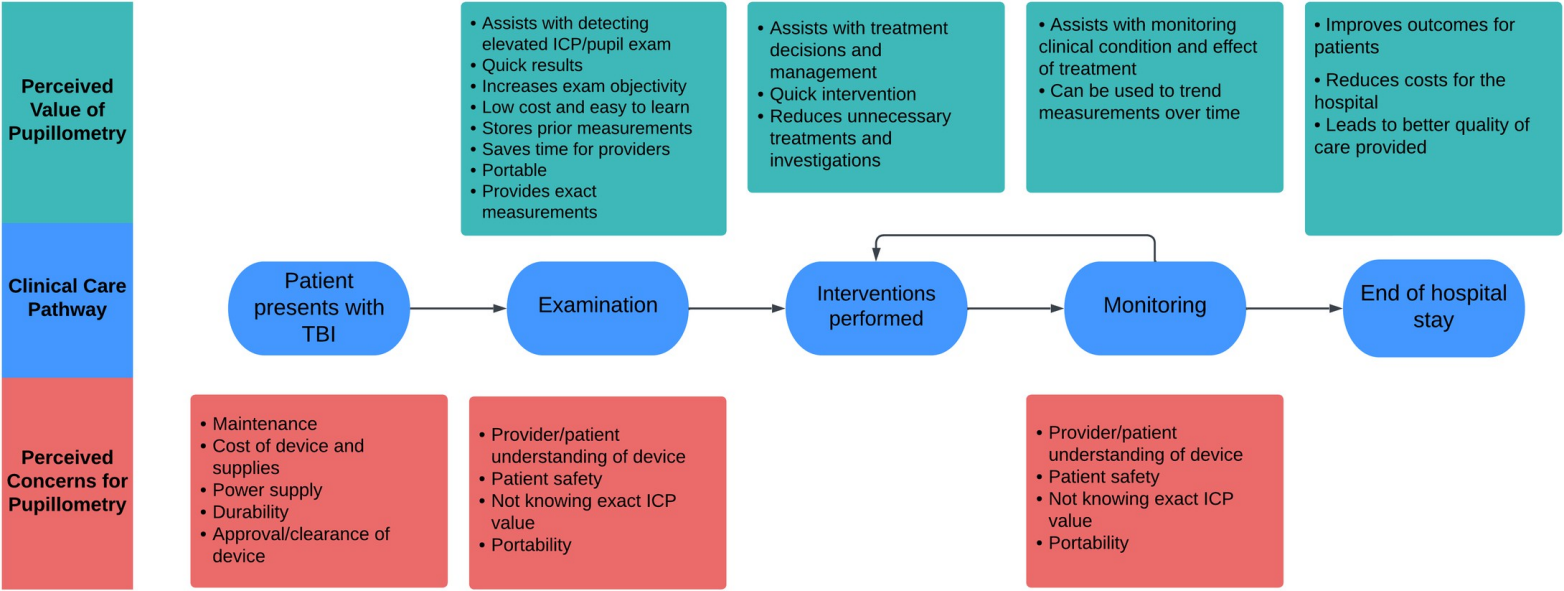
Perceived value of pupillometry and concerns for pupillometry across the clinical care pathway.

In both interviews, participants discussed characteristics of the pupillometer that add value to the care of patients with TBI (Table 2) along different points of the clinical care pathway, with frequencies shown in Table 2. The discussion points were categorized according to whether they addressed the impact on examining patients, delivering interventions, monitoring patients, and/or overall care results seen at discharge (Fig 2). The most commonly mentioned way the pupillometer would add value on the initial exam was by detecting elevated ICP. An emergency ward neurosurgeon described this impact:

**Table 2:** Frequency of themes related to value of pupillometry and concerns for pupillometry and when the theme was discussed*.

Topic	Theme	Frequency (n, %)	Interview (Pre-training, post-training, or both)
Value	Assists with treatment decisions/management	21, 95%	Both
	Detecting elevated ICP	19, 86%	Both
	Quick intervention	17, 77%	Both
	Quick results	17, 77%	Both
	Pupil exam	12, 54%	Both
	Monitoring	11, 50%	Both
	Improved outcomes	10, 45%	Both
	Reduces interobserver variability	10, 45%	Both
	Low cost	6, 27%	Both
	Assists with monitoring of treatment effect	5, 23%	Both
	Better quality of care provided	5, 23%	Both
	Reduces costs	5, 23%	Post-training
	Reduces unnecessary treatments	5, 23%	Both
	Reduces investigations performed	5, 23%	Both
	Easy to learn	4, 18%	Both
	Stores previous measurements	4, 18%	Both
	Saves time for providers	3, 14%	Post-training
	Trending measurements	3, 14%	Pre-training
	Exact measurements	2, 9%	Post-training
	Portability	2, 9%	Post-training
Concerns	Maintenance	14, 64%	Both
	Understanding of device	13, 59%	Both
	Cost of device	12, 55%	Post-training
	Durability	7, 32%	Both
	Patient safety	6, 27%	Both
	Not knowing exact ICP value	4, 18%	Pre-training
	Cost of SmartGuard®	4, 18%	Both
	Approval/clearance	3, 13%	Both
	Portability	3, 14%	Both

*Only themes that were discussed by 2 or more participants were included. Refer to S6 Table in S1 File for additional themes.

This tool will help us pick more of those patients who don’t show clear signs of raised intracranial pressure, those with hidden signs. I believe, and I hope, that we shall be able to catch more of them and do more investigations earlier and therefore help them, treat them promptly.

When discussing patient intervention, pupillometry was most commonly described as adding value through treatment decisions and patient management. A neurosurgeon described the impact a pupilometer would have in decision-making regarding patient interventions, stating,

It would allow me to manage ICP better and make better clinical decisions in a timely fashion, basically. So I would be able to make fast, quicker decisions on who to operate, who not to operate, who to change medication, […] who needs a repeat CT scan.

As patients remain in the hospital and receive monitoring, the most commonly described role of pupillometry was monitoring the patient’s clinical condition. At discharge, the most commonly discussed value of pupillometry was how it could improve patient outcomes, including reducing hospital length of stay and decreasing rates of mortality.

Specific concerns related to pupillometry were also discussed (Table 2) and categorized according to their need to be addressed at different stages of the clinical care pathway (Fig 2). Identified concerns would optimally be addressed before a patient arrives at the hospital or during the examination and monitoring of patients. Of the concerns that would need to be addressed before patient arrival, the most commonly discussed were the cost of the pupillometer and device maintenance. One neurosurgeon related their concerns of cost to their experiences with other machines used in their setting, stating, “The cost. […] We live in a setting where you will fail to find a blood pressure machine, so asking for a pupillometer is a bit of too much to ask.” While not discussed during the education session, the estimated cost of obtaining a pupillometer, charging device, and SmartGuards® to perform pupillometry exams for 24 patients would be just less than $7000 USD. This may be a significant cost at MNRH, however, it is important to consider that this is lower than the cost of invasive monitoring. Regarding maintenance, a general surgery resident related their concerns to previous experiences with new technology, stating, “We have seen many other devices being bought from other countries and when we are using it, you don’t, get the spare part, can’t find the spare part. No one knows how to do servicing on it.” Of the concerns that would need to be addressed during examining and when monitoring patients, the most commonly discussed was the provider and patient understanding of the device.

Participants also discussed the ways that pupillometry might impact their clinical work. While not specifically prompted, nine participants (41%) mentioned that pupillometry might decrease their workload. When asked how pupillometry might impact the time they spend with patients, 13 participants (59%) thought that pupillometry would decrease the time they spent with patients. However, three participants (14%) thought using the pupillometer would increase the time they spend with patients. Other traits of pupillometry discussed included accuracy, helpfulness, reliability, and trust. Most participants stated that they thought the pupillometer would be accurate (n = 15, 68%) and helpful (n = 17, 77%). While not prompted, five participants (23%) described the pupillometer as reliable. Additionally, 12 participants (55%) described wanting to use the pupillometer in their clinical work, and three participants (14%) described wanting the use of the device extended to additional locations. Participants also discussed expected perceptions of pupillometry by patients and their colleagues. Most participants thought that patients (n = 14, 64%) and other providers (n = 17, 77%) would positively perceive pupillometry.

In the post-training interview, participants were asked about their overall perception of the device and monitoring preferences. Of the 19 participants, 15 (79%) stated they had a positive overall perception. No participants reported having an overall negative perception. Preferences for monitoring patients with TBI varied. Five participants (26%) reported having no preference, five (26%) preferred noninvasive monitoring, five (26%) preferred pupillometry specifically, and three (16%) preferred invasive monitoring.

### Implementation of pupillometry

At the beginning of the pre-training interview, participants discussed the current care environment for patients with TBI at MNRH. Participants identified factors that currently influence decision making for patients with TBI, including the clinical status of the patient (n = 11, 50%), availability of needed resources (n = 4, 18%), the risks and benefits of different treatment options (n = 2, 9%), and imaging results (n = 8, 36%). When discussing current ICP monitoring practices, 17 participants (77%) described the use of clinical assessment for monitoring, and eight participants (36%) mentioned that they don’t currently have any devices to use for monitoring. Two participants (9%) described invasive monitoring in select cases. For example, a neurosurgeon described the use of EVD in the ICU:

Then in the ICU, when we are able we put in EVDs, […] we may be able to measure the ICP, intracranial pressure, using column of, a column of water and a drainage tube. So we measure it with that and we are able to determine the pressure at intervals. It is not a continuous thing. And we are also able to drain CSF and treat the intracranial pressures in these patients. We don’t do that routinely.

Most participants stated that after elevated ICP is suspected in a patient, providers proceed with treatment through conservative management (n = 16, 73%) or surgical intervention (n = 15, 68%).

When considering the implementation of pupillometry, nine participants (41%) thought that implementation would be easy in their setting, while two (9%) thought implementation would be difficult. After completing training, five participants (23%) reported that the pupillometer was easy to use, and one participant (5%) described the device as difficult to use. Five participants (23%) thought the device was initially difficult to use but became easier during the practice session.

Participants also discussed various aspects of ideal device use. When considering the timing of the pupillometer exam, most participants recommended repeating pupillometry exams regularly (n = 17, 77%) and/or adjusting the timing of use based on a patient’s clinical condition (n = 14, 64%). Others recommended use of the device immediately upon a patient’s presentation to the hospital (n = 8, 36%), at certain times of day (n = 3, 14%), or during every encounter with a patient (n = 2, 9%). Participants also discussed the types of providers that they thought should use pupillometry. Many participants (n = 12, 55%) recommended that only healthcare workers involved in the clinical care of patients or those who received training should use pupillometry. The types of providers that participants recommended use pupillometry included doctors (n = 15, 68%), nurses (n = 12, 55%), residents (n = 9, 41%), medical officers (n = 8, 36%), interns (n = 4, 18%), and students (n = 2, 9%). Some participants (n = 10, 45%) described wanting to use pupillometry in patient populations outside of patients who have TBI.

**Fig 3:**
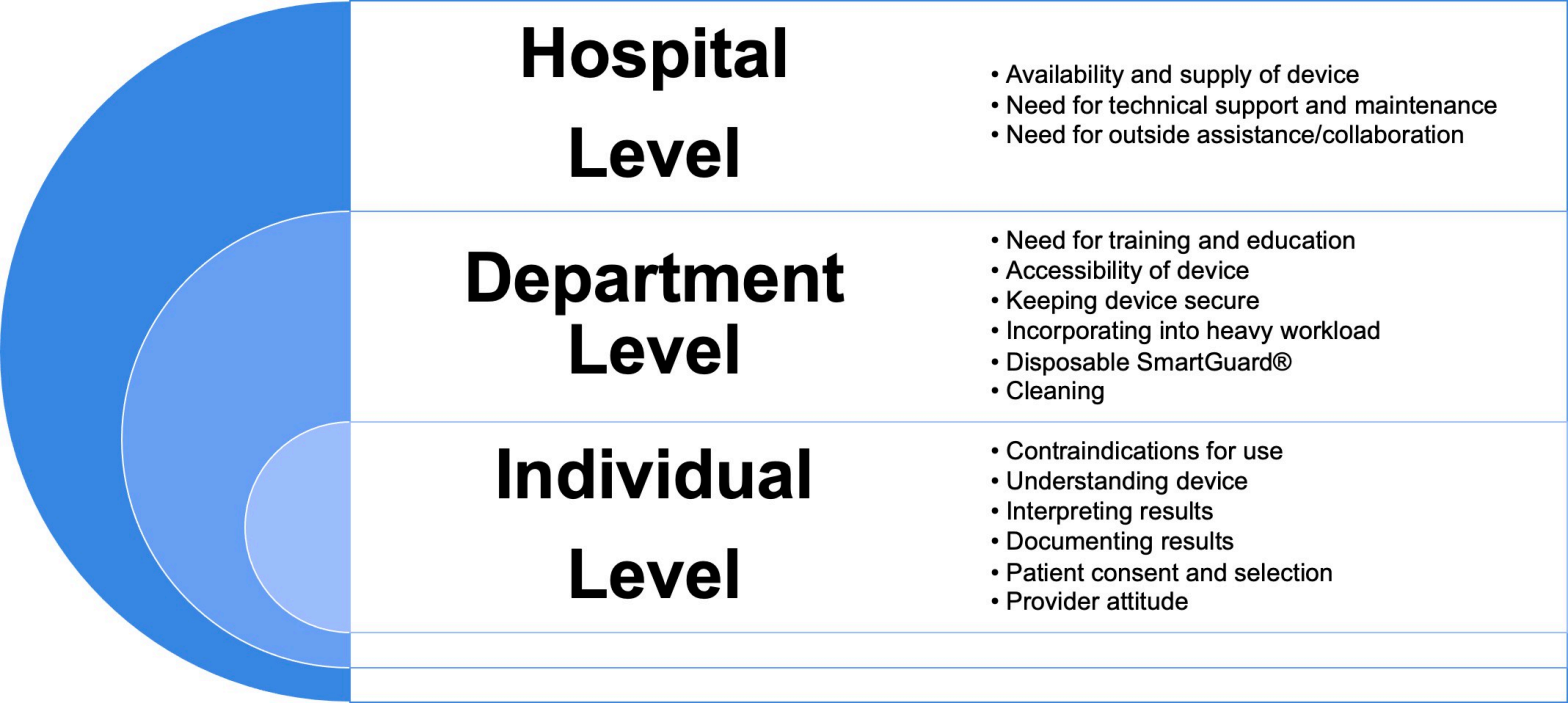
Perceived challenges for implementing pupillometry at the hospital level, department level, and provider level.

Participants identified many potential challenges for implementing pupillometry in their setting (Table 3). These challenges were organized based on impact at the hospital, department, and individual patient/provider levels (Fig 3). At the hospital level, the availability of the pupillometer and related supplies was the most commonly mentioned challenge. At the department level, the need for further education and training was the most commonly described challenge. At the individual level, the most common challenge discussed was possible patient contraindications for pupillometry. A neurosurgeon gives an example of this difficulty, stating,

**Table 3:** Frequency of themes related to implementation challenges and implementation strategies and when the theme was discussed*.

Topic	Theme	Frequency (n, %)	Interview (Pre-training, post-training, or both)
Challenges	Availability and supply of device	19, 86%	Both
	Need for further training	9, 41%	Both
	Contraindications	7, 32%	Both
	Need technical support or maintenance support	6, 27%	Both
	Provider understanding of device	6, 27%	Post-training
	Accessibility of device	5, 23%	Both
	Keeping device secure	5, 23%	Both
	Interpreting results	4, 18%	Both
	Incorporating into heavy workload	3, 14%	Post-training
	Documentation of results	3, 14%	Both
	Disposable SmartGuard®	3, 14%	Post-training
	Cleaning	3, 14%	Post-training
	Provider attitude	2, 9%	Post-training
	Need outside assistance/collaboration	2, 9%	Both
	Patient selection	2, 9%	Post-training
Strategies	Continued education	19, 86%	Both
	Hospital support	11, 50%	Both
	Providing technical/maintenance support	7, 32%	Both
	Designate team or someone to oversee	7, 32%	Post-training
	Make devices available	6, 27%	Post-training
	Ensure patient understanding	6, 27%	Both
	Incorporate into workflow	4, 18%	Both
	Safe storage	4, 18%	Post-training
	Education manual	3, 14%	Pre-training
	Promote staff ownership	2, 9%	Pre-training
	Supply/procurement plan	2, 9%	Post-training

*Only themes that were discussed by 2 or more participants were included. Refer to S7 Table in S1 File for additional themes.

The challenge of […] not being able to use this device in patients who have local trauma to the eye and the nerves, optic nerve, and the oculomotor nerve. And, of course, you have to have a cooperative patient or a comatose patient, so those restless patients may not be easy to work with.

**Fig 4:**
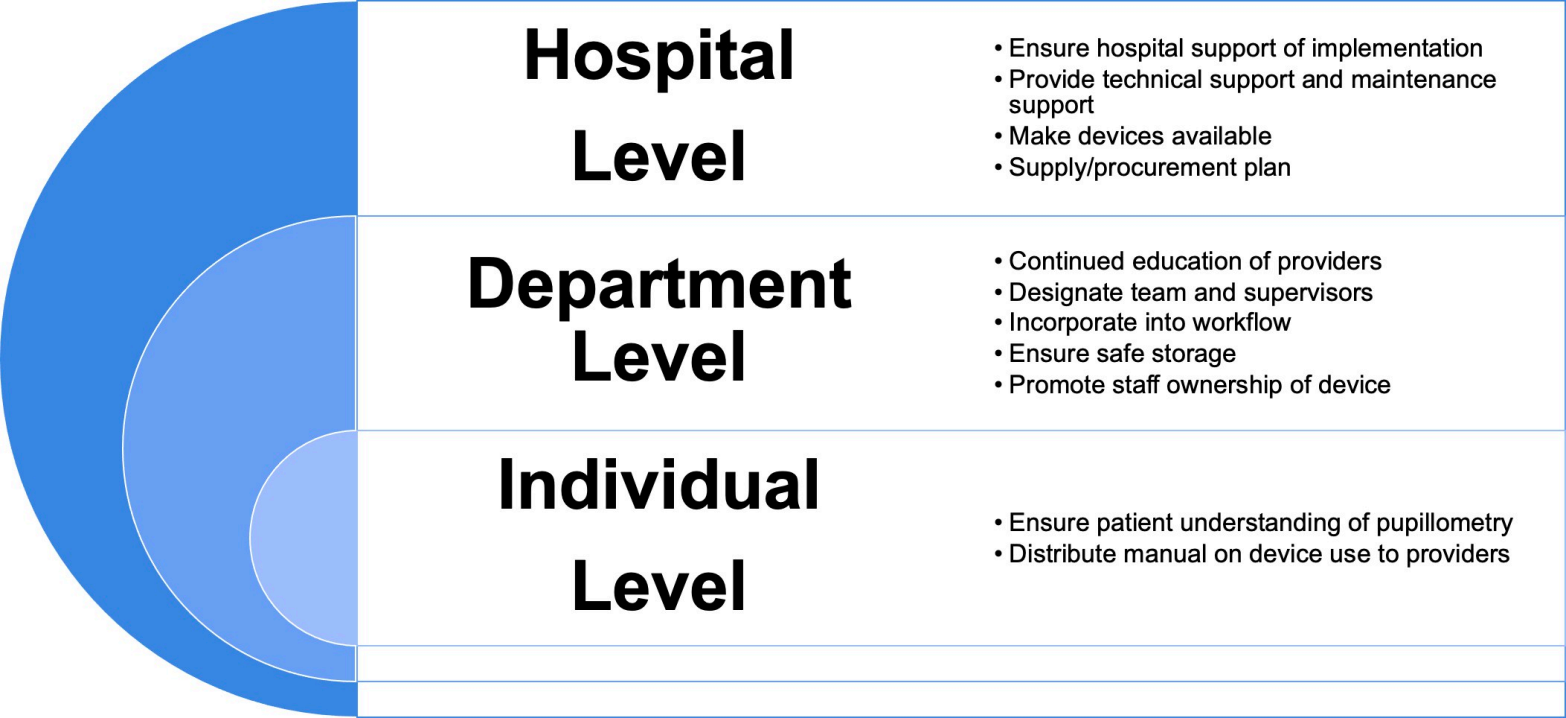
Proposed strategies for successful implementation of pupillometry at the hospital level, department level, and provider level.

Alongside the potential challenges identified, participants suggested multiple strategies that could aid in successfully implementing pupillometry in their setting (Table 3), which were organized based on the need for action at the hospital, departmental, and individual patient/provider levels (Fig 4). At the hospital level, ensuring support from hospital administrators was the most commonly recommended strategy. At the department level, organizing continued education around pupillometry was the most commonly described strategy. At the individual level, participants most frequently recommended ensuring patient understanding of pupillometry, as described by a neurosurgery ward nurse who stated, “I think we have to give health education and, even before, we need to explain [to] the patient if the patient is in the state of understanding. If not, […] we can explain to the caretakers.”

### Fidelity of pupillometry measurement

During the training session, the average time to learn to use the pupillometer and obtain 16 practice measurements was 13.5 minutes (IQR = 3.5). The average time to obtain a measurement of both eyes using the pupillometer in the training session was 50.6 seconds (IQR = 11.8). For the right and left eye measurements of NPi®, 21 participants (100%) had comparison measurements within the expected clinical range of 0.5. For the left and right eye measurements of pupil size, two participants (9.5%) had measurements within the expected range of 0.03mm. Using the expected clinical range of 0.5, 19 participants (90.5%) had comparison measurements within the expected range for the left eye, and 21 participants (100%) had comparison measurements within the expected range for the right eye (Table 4).

**Table 4:** Fidelity measurements from pupillometry training session.

Category	Fidelity Measurement	Average (IQR)	
Learning measurements	Time to learn (minutes)	13.5 (3.5)	
	Time to obtain measurement (seconds)	50.6 (11.8)	
	Fidelity Measurement	Count (%)	
Measurements within expected range	Left eye NPi®	21 (100%)	
	Right eye NPi®	21 (100%)	
	Left eye pupil size (device error)	2 (9.5%)	
	Right eye pupil size (device error)	2 (9.5%)	
	Left eye pupil size (clinical)	19 (90.4%)	
	Right eye pupil size (clinical)	21 (100%)	
	Fidelity Measurement	Mean, variance	P-Value
Accuracy of Measurement	Participant left eye NPi®	4.262, 0.113	0.437
	Trainer left eye NPi®	4.238, 0.142	
	Participant right eye NPi®	4.243, 0.146	0.916
	Trainer right eye NPi®	4.238, 0.099	
	Participant left eye pupil size	3.329, 0.667	0.944
	Trainer left eye pupil size	3.337, 0.510	
	Participant right eye pupil size	3.346, 0.427	0.483
	Trainer right eye pupil size	3.309, 0.351	

## Discussion

This study provides a novel understanding of the feasibility of training and potential use of pupillometry for ICP monitoring in a national referral hospital in Uganda. Through qualitative interviews with providers, this study identified ways that pupillometry could be beneficial in the care of patients with TBI, concerns providers could have regarding pupillometry, expected challenges that could be faced during implementation, and potential strategies to aid in implementation. Additionally, this study found that training with the device was quick and led to adequate use by participants on healthy volunteers by the end of a training session.

### Perception of pupillometry

This study found a mostly positive perception of pupillometry among healthcare workers at MNRH and identified potential strengths of pupillometry if it were to be used as a method of noninvasive monitoring. Participants described the ways that pupillometry could be valuable in the care of patients with TBI at multiple steps of the clinical care pathway, including how pupillometry could assist with performing the pupil exam, detecting elevated ICP, increasing objectivity of the neurological exam, making treatment decisions, and monitoring a patient’s clinical condition. They also discussed how pupillometry could be useful for other patients at risk of having elevated ICP. Participants also described how they thought pupillometry could lead to the provision of a better quality of care and improved outcomes for patients. Additionally, most providers reported thinking that pupillometry was accurate and helpful. These findings are in agreement with existing literature on the advantages of pupillometry in patients with TBI, including its association with elevated ICP, [18, 20–24] reduced intraobserver and interobserver variability compared to the manual pupil exam, [14, 16] and quick detection of changes in the pupil response [14].

This prior research, along with these study findings, suggests that pupillometry could be beneficial as a method of noninvasive monitoring for patients with TBI; however, no prior studies on the clinical use of pupillometry for noninvasive ICP monitoring in any setting were identified. Reasons for this could include a preference for invasive monitoring when it is accessible and a reduced need for alternative monitoring strategies in HICs where invasive monitoring is widely available. Additionally, no studies on the use of pupillometry for any purpose in any LMIC settings were identified. While pupillometry is considered low cost compared to other ICP monitoring strategies, difficulty obtaining funding for start-up costs of pupillometry and limited knowledge of pupillometry could be additional factors contributing to why pupillometry has not yet been used in LMICs.

Importantly, this study identified concerns related to pupillometry, including concerns specific to MNRH and other limited-resource settings. In studies of the implementation of pupillometry in HIC settings, concerns like access to a power supply, maintenance availability, device durability, and cost of the pupillometer and supplies have not been described as implementation barriers [16, 32–34]. These concerns identified in this study could be related to barriers faced in the neurosurgical system in Uganda, including unreliable access to electricity, [35] difficulty with the maintenance of imported technology, [36, 37] and limited health system funding [38].

Another concern identified in this study likely relates to the challenge of using pupillometry in LMICs, where alternative, invasive methods of ICP monitoring are often inadequate or unavailable [11]. While many providers in this study discussed the value of pupillometry to detect elevated ICP, some expressed concern that pupillometry could not be used to gain an exact, numerical estimate of ICP. This is a limitation of pupillometry that would need to be accepted by providers for its use.

Other concerns identified likely relate to the novelty of pupillometry in the study setting. Participants described concern over the approval of the device, patient safety, and the ability of providers and patients to understand pupillometry. Given that pupillometry has been safely implemented in other settings without adverse effects reported, [16] efforts to increase knowledge and increase awareness of the device among patients, providers, and healthcare administrators could help address these concerns.

### Implementation of pupillometry

This study identified potential barriers and strategies that are similar to those reported with the implementation of pupillometry in HIC settings. The most commonly described implementation challenge in this study was an expected difficulty in obtaining an adequate supply of pupillometry devices and associated consumables to ensure availability. Similarly, in a neurocritical care unit in the US, the availability of pupillometers after implementation was found to be a challenge for the use of pupillometry, and increasing device availability increased compliance with use [39]. The availability of SmartGuards® has also been reported as a challenge in the implementation of pupillometry in HIC settings [34]. Along with the availability of pupillometry devices, this study identified the storage of devices in a safe and easily accessible location as another potential barrier to implementation. Ensuring hospital support of the implementation of pupillometry and creating supply and procurement plans were strategies suggested by participants in this study that could address issues concerning device availability and access.

Participants in this study also discussed the need for the education of providers. Provider knowledge has also been cited as a barrier to use in Australia, [34] suggesting that education and training are important components of pupillometry implementation. Concern over documenting results obtained from pupillometry was also discussed as a barrier to implementation. Documentation of results was similarly found to be a barrier to using pupillometry in previous studies in Australia and the US [32, 34]. While integrating pupillometry results into the electronic medical record (EMR) led to improved use in one of these studies, [32] alternative strategies would be needed to address this challenge at MNRH, where an EMR is not currently used.

Another identified consideration for the implementation of pupillometry in this study was the need for technical and maintenance support. In LMICs, inadequate consideration of maintenance needs and costs contributes to challenges with inadequate use of medical devices after procurement [40]. At MNRH, an existing collaboration with Duke Global Neurosurgery and Neurology has improved access to technology in the neurosurgery department. Strategies that have helped ensure the successful introduction of new technology through this collaboration have included consideration of the appropriateness of equipment before its donation, along with training and follow-up for local clinical engineers to meet maintenance needs [36, 37, 41]. While this study is the first step in assessing the appropriateness of the introduction of pupillometry at MNRH, further efforts to implement pupillometry would require the involvement of biomedical technicians.

Additionally, providers discussed their recommendations for the use of pupillometry. Given that abnormal NPi® is associated with elevated ICP, [20–24] participants discussed how pupillometry could be implemented for detecting new elevation in ICP. As discussed by participants, pupillometry could be used in patients with TBI upon initial presentation, and then repeated to assess for changes in the pupil exam. Participants discussed that the timing of the pupillometry exam would need to take a patient’s clinical status into consideration, and the authors did not identify more specific guidelines on the recommended timing for the pupillometry exam. Considering the participants’ recommendations, the implementation of pupillometry could include completion of the pupillometry exam by clinical providers like nurses, residents, and neurosurgeons at regular intervals depending on the patient’s location and clinical status. Information obtained from pupillometry could be incorporated into existing clinical decision making and treatment algorithms. Abnormal pupillometry results could inform decisions to begin medical management or obtain repeat imaging. Because pupillometry can detect changes in the pupil exam more quickly than the clinical pupil exam, it was thought that pupillometry could alert providers to a concerning change in clinical status and prompt a response sooner.

### Pupillometry fidelity

Fidelity measurements taken during the training session demonstrated that the time to learn pupillometry was short. Providers could complete 16 practice measurements on healthy volunteers in an average of 13.5 minutes. Additionally, the average time to take a measurement during the training session was 50.6 seconds. Given that this estimate was obtained during training, this suggests that pupillometry could be quick to use in the study setting if implemented. These findings are consistent with those from a previous study which estimated that the time for a trained provider to complete a set of pupillometry measurements was 37 seconds [32]. While the findings from this previous study were obtained after the implementation of pupillometry and included the time to document results in its estimate, the results of this study suggest that even during training, pupillometry measurements were quick to obtain.

Additionally, this study found that participants could obtain relatively accurate measurements on healthy volunteers. Paired t-tests indicated no statistical difference in measurements of NPi® and pupil size obtained by the participant after training compared to measurements obtained by the trainer. Most participant measurements were within the clinically significant cut-off of 0.5 units of the trainer’s measurement for NPi® and pupil size. This study also assessed whether any differences in measurements between users were within the expected device error, which has not been assessed in prior studies. While most paired pupil size measurements were not within the device error cutoff of 0.03mm, these differences likely would not impact clinical decision-making.

### Study strengths and limitations

This study is one of the first to assess the perception and feasibility of pupillometry for noninvasive monitoring in an LMIC. While this study focused on just one hospital in Uganda, its findings can inform efforts to use pupillometry worldwide. Differences and similarities in the perception of pupillometry and its implementation were identified in this study compared to findings from studies in HIC settings. Additionally, the inclusion of providers from multiple roles provided a better understanding of perspectives of pupillometry and a more holistic evaluation of provider perspectives.

There are important limitations of this study to consider. Purposive sampling was used to recruit participants for the study, which could have led to an overrepresentation of providers interested in learning about a new technology. Thus, this study may not have captured the views of as many people with hesitations about using such a technology. Additionally, all data were collected by a student from a HIC university with a history of collaboration with MNRH. This may have led to a power imbalance between the researcher and participants and may have impacted the opinions that participants were willing to share during their interviews. This student also was the sole researcher involved in the education, training, and interview sessions; thus, this may have generated some bias in responses during individual interviews as participants may be less willing to share negative perceptions considering this close relationship. This study also only assessed perception and feasibility based on theoretical knowledge and limited practical use of pupillometry, so actual perception and feasibility could vary if pupillometry were integrated into patient care. It would be necessary to study the perception and feasibility of pupillometry use in patients with TBI, because additional challenges may exist that were not seen when the device was used with healthy volunteers. Patients with TBI may have injuries affecting the orbits or cranial nerves and may be less cooperative with undergoing a pupillometry exam, and additional challenges may exist with differing light environments in clinical settings. This study also did not discuss the use of pupillometry in the ICU, so further studies would be needed to assess the feasibility of the use of pupillometry in this setting. Finally, there were varying levels of knowledge obtained from the education session as evaluated during the interviews. While the interviews were not standardized to assess provider knowledge, differences in understanding of pupillometry and its relation to elevated ICP may have impacted participants’ views.

## Conclusion

Through qualitative interviews on the perception of pupillometry and its implementation, as well as quantitative fidelity measurements obtained during a training session, this study evaluates the feasibility of training and perception of the potential use of pupillometry for noninvasive ICP monitoring at a national referral hospital in Uganda. Most participants reported a positive perception of pupillometry. It was thought that pupillometry could add value at multiple steps in the clinical care pathway for patients with TBI, including supporting treatment decision-making, allowing for quick intervention, helping monitor patients’ clinical condition, and improving patient outcomes. Concerns were also identified, including the cost of pupillometry, required maintenance, and patient and provider understanding of the device. Potential challenges and strategies for implementing pupillometry into clinical practice were also discussed, including availability and accessibility of the device, providing education for providers, and contraindications for use. Suggested strategies to promote the successful implementation of pupillometry included ensuring support from hospital administrators, providing continued education on pupillometry, and promoting provider and patient understanding. Additionally, findings from the training session suggest that pupillometry training would be feasible. However, the concerns, implementation challenges, and strategies for the implementation of pupillometry identified in this study would need to be considered if pupillometry were to be used in the study setting in the future. This study was one of the first to assess the potential use of pupillometry in an LMIC setting and allowed for identification of barriers to the use of pupillometry that may reflect differential challenges that could be faced in such a setting. Future work could assess the actual perception and feasibility of pupillometry if implemented and evaluate whether the use of pupillometry for noninvasive ICP monitoring leads to clinically meaningful benefits.

## Supporting information

S1 File (DOCX)
