# Supplementary material for: Exploring the feasibility of pupillometry training and perceptions of potential use for intracranial pressure monitoring in Uganda: A mixed methods study
Source: PLoS One. 2024 May 15;19(5):e0298619. doi: 10.1371/journal.pone.0298619 (PMC11095748; doi:10.1371/journal.pone.0298619)
Supplement: S1 File — (DOCX) [file pone.0298619.s001.docx]

**S1 Interview Guides**

## Pre-Training Interview Guide

The pre-training interviews in this study will all cover the same topics but will do so organically. Interview questions may not be phrased in the exact same way in each interview, and they may not progress in the same order. This interview guide contains questions that cover the key topics that will be discussed during each interview. It is possible that interviewees will ask follow up questions that cover unanticipated topics. Emergent topics will be included at the discretion of the researcher and may be incorporated into future interviews if they are relevant to the study.

**Introduction**

Hello, my name is _________ Today, I want to talk to you about your perspectives on noninvasive methods of measuring intracranial pressure as discussed in the education session. I will be asking questions about your perspective about ICP measurement, opinions on the device showcased, and experiences implementing new technology into your workflow. All of your responses are valuable, so please feel free to share whatever you think is important. I’m here to learn about your thoughts, whatever they may be. We will be recording this discussion and the microphone will be taping the session so we do not miss any important information.

Do you have any questions before we begin?

**Demographic Information**

I would like to start by gathering some demographic information about you

How old are you?

What is your gender?

What is your role in the hospital?

Where did you train before starting your role?

How long have you been in practice?

Do you work full time or part time?

If part time: Do you have any other jobs

Do you work directly with patients in your job?

If no: who do you interact with daily in your job?

|  | | |
| --- | --- | --- |
| **Parent Question** | **Focus of Question** | **Probes** |
| *Part 1- First, I want to get a general sense of your experiences with treating traumatic brain injury here at the hospital. Just answer these questions as fully as you can and if you’re not sure about a question, please don’t hesitate to ask for clarification.* | | |
| When did you start working for the hospital? | To build rapport; to ease the participant into talking about experiences |  |
| How often do you see or treat patients with traumatic brain injury? | To get a better understanding of participant’s personal experiences with treating the disease |  |
| Please tell me about your role in treating patients with traumatic brain injury. | To get a better understanding of participant’s personal experiences with treating the disease |  |
| What is your understanding of how treatment decisions are made for patients with traumatic brain injury? | To get a better understanding of participant’s personal experiences with treating the disease | Who makes the treatment decisions? |
| *Part 2- For this next set of questions, I want to talk with you about your understanding of noninvasive intracranial pressure monitoring and about the technology you learned about in the education sessions. We know that you have not had a chance to get familiar with the technology yet, so again, it’s okay if you don’t know the answers, and if something isn’t clear, please let me know and ask any questions you need to.* | | |
| To start, can you explain to me the importance of intracranial pressure monitoring for a patient with traumatic brain injury? | To get an understanding the participant’s knowledge surrounding ICP and TBI | If prompting needed: what does an elevated intracranial pressure mean? Is it associated with a good or bad outcome? |
| How do the clinicians in the hospital currently monitor for intracranial pressure in patients with traumatic brain injury? | To get an understanding of participant’s knowledge of current hospital practices |  |
| When a clinician suspects elevated intracranial pressure in a patient, what do they do next? | To get an understanding of participant’s knowledge of current hospital practices | Why do they treat the patient in this way? |
| *Now we will discuss the technology that was presented at the education session.* | | |
| In your own words, can you explain to me the pupillary light reflex? | To investigate previous understanding or knowledge obtained from education session |  |
| How does the pupillary light reflex relate to intracranial pressure? | To investigate previous understanding or knowledge obtained from education session |  |
| What tool was presented to examine pupillary light reflex? | To investigate knowledge obtained from education session | Can you please describe the tool?  What does the tool do?  When should the tool be used?  How long would it take to use the tool to get a measurement? |
| How do the readings from the pupillometer relate to intracranial pressure? | To investigate knowledge obtained from education session |  |
| What is your opinion on using this tool to monitor intracranial pressure? | To obtain the participant’s opinion about the device | Do you have any concerns using this tool?  How accurate, or inaccurate, do you think the readings from the device are likely to be?  How helpful, or not helpful, do you think the readings from the device are likely to be? |
| *Part 3- For this third set of questions, I would like us to discuss how you think using this new technology will impact your workflow and patient care.* | | |
| Do you think the proposed technology will change the amount of time needed to be spent with TBI patients? | To obtain the participant’s opinions about utilizing device in patient care | In what way will the amount of time spent with patients change? |
| In what ways do you think using the pupillometer device would benefit your clinical practice? | To obtain the participant’s opinions about utilizing device in patient care | What about the device makes you say that? |
| In what ways do you think this tool would have negative effects on your clinical practice? | To obtain the participant’s opinions about utilizing device in patient care | What about the device makes you say that? |
| How plausible do you think it is to incorporate using this device into your patient workflow as described in the education session? | To obtain the participant’s opinions about utilizing device in patient care | How might you incorporate this device into your workflow? |
| In your opinion, what is the ideal patient scenario for using this device? | To obtain the participant’s opinions about utilizing device in patient care |  |
| In your opinion, how often would it be ideal to monitor patients with this device? | To obtain the participant’s opinions about utilizing device in patient care |  |
| *Finally, do you have any questions or anything else you’d like to add?*  *Thank you for taking the time to speak with us today* | | |

#

Post-training Interview Guide

The post-training interviews in this study will all cover the same topics but will do so organically. Interview questions may not be phrased in the exact same way in each interview, and they may not progress in the same order. This interview guide contains questions that cover the key topics that will be discussed during each interview. It is possible that interviewees will ask follow up questions that cover unanticipated topics. Emergent topics will be included at the discretion of the researcher and may be incorporated into future interviews if they are relevant to the study.

**Introduction**

Hello, my name is _________ Today, I want to talk to you about your perspectives on the noninvasive method of measuring intracranial pressure that has been discussed as a part of this research study. I will be asking questions about your perspective about the device. All of your responses are valuable, so please feel free to share whatever you think is important. We will be recording this discussion and the microphone will be taping the session so we do not miss any important information. Do you have any questions before we begin?

**Demographic Information**

I would like to start by gathering some demographic information about you

How old are you?

What is your role in the hospital?

Do you work full time or part time?

If part time: Do you have any other jobs

Do you work directly with patients in your job?

If no: who do you interact with daily in your job?

| **Question** | **Focus of Question** | **Probes** |
| --- | --- | --- |
| *Part 1- First, I want to get a general sense of your experiences with treating traumatic brain injury here at the hospital. Answer these questions as fully as you can and if you’re not sure about a question, please don’t hesitate to ask for clarification.* | | |
| In the period of time since you completed the first interview, has your role with treating TBI patients changed? | To get a better understanding of participant’s background with treating the disease |  |
| When were you first introduced to the concept of noninvasive ICP monitoring? | To build rapport; to ease the participant into talking about experiences |  |
| How do you think TBI patients could benefit from noninvasive ICP monitoring devices? | To get a better understanding of participant’s beliefs about the device’s utility |  |
| *Part 2- Pupillometer: We will now discuss implementing the pupillometer.* | | |
| What barriers could exist for implementing pupillometry here at the hospital? | To assess perceived barriers for the implementation of pupillometry |  |
| What strategies could help with successful implementation of pupillometry? | To assess perceived facilitating factors for the implementation of pupillometry |  |
| During training, how easy or difficult was it to use the pupillometer? | To assess perception of ease of use of pupillometry after completion of training |  |
| In your opinion, what would your colleagues and coworkers' attitudes be towards pupillometry? | To assess perceived or observed interprofessional sentiments regarding ICP monitoring methods. | Why do you believe they will feel this way? |
| In your opinion, how do you believe patients will perceive the usage of the pupilometer? | To assess perceived or observed patient sentiments regarding ICP monitoring methods. | Why do you believe they will feel this way? |
| In your opinion, would the data you obtain from the pupillometer change the way you deliver patient care? | To evaluate effects on patient care. | If yes, how would it change the way you treat patients? |
| In your opinion, what role or combination of roles in the hospital should be responsible for obtaining these measurements (examples: nurses, physicians, medical officers, etc.)? | To learn about ideal workflow from someone with interprofessional insight. | Why do you think this is best? |
| What is your perception of pupillometry measurement for ICP monitoring? | To understand the overall perspective of noninvasive monitoring methods. |  |
| Do you have any preference for noninvasive ICP monitoring or other forms of ICP monitoring? If so, what is your preference? | To understand the overall perspective of noninvasive monitoring methods. | What are the reasons for this preference (or lack of preference)? |
| *Finally, do you have any questions or anything else you’d like to add?*  *Thank you for taking the time to speak with us today* | | |

**S2 Checklist: Inclusivity in Global Research**

**Ethical considerations, permits and authorship**

Provide details as to who granted permissions and/or consent for the study to take place in the Methods section of your manuscript. This should include the names of **all** ethics boards, governmental organizations, community leaders or other bodies that provided approval for the study. If individuals provided approval refer to these people by their role or title but do not list their name(s).
Duke University Hospital IRB and Mulago National Referral Hospital reviewed and approved this study. The study was also approved by the head of Neurosurgery at Mulago National Referral Hospital.

If there were any deviations from the study protocol after approval was obtained please provide details of these changes in the Methods section of your manuscript.
N/A

Did this study involve local collaborators that are residents of the country where the research was conducted or members of the community studied? If you do not have any authors from said communities, please provide an explanation for this below.

Yes, this study involved local collaborators in study planning and implementation.

Everyone listed as an author should meet PLOS’ criteria for authorship and all individuals who meet these criteria should be included in the author byline, rather than the acknowledgements. Authorship criteria is based on the International Committee of Medical Journal Editors (ICMJE) Uniform Requirements for Manuscripts Submitted to Biomedical Journals - for further information please see here: <https://journals.plos.org/plosone/s/authorship>.

**Human subjects research (e.g. health research, medical research, cross-cultural psychology)**

Did you obtain written informed consent from a representative of the local community or region before the research took place? How did you establish who speaks for the community? Details of written informed consent obtained from study participants should be reported separately in the Methods section of your manuscript.

N/A

How did members of the local community provide input on the aims of the research investigation, its methodology, and its anticipated outcome(s)? How did members of the local community provide input on the aims of the research investigation, its methodology, and its anticipated outcome(s)?

Members of the local community were involved in planning meetings to determine the aims, methodology, and anticipated outcomes of the research.

When engaging with the local community, how did you ensure that the informed consent documents and other materials could be understood by local stakeholders?

The study only involved healthcare providers who all spoke English, the language used in the study.

Will the findings of the research be made available in an understandable format to stakeholders in the community where the study was conducted (e.g. via a presentation, summary report, copies of publications, etc.)? Please provide details of how this will be achieved.

The results of the study will be delivered to the community stakeholders via presentation once the results have been published.

**Non-human subjects research using specimens/ animals collected as part of the study, or those housed in archival collections. Examples include archaeology, paleontology, botany and zoology.**

Did the permission you obtained from a local authority to perform the study include an agreement on access to outputs and benefit sharing? This may include procedures to enable fair distribution of the benefits and resources arising from the research performed. Please include any details of Prior Informed Consent and Benefit Sharing Agreements obtained. These may be required by field-specific regulations, for example the Convention on Biological Diversity (CBD) and the associated Nagoya Protocol.

N/A

If the material used in your study was imported, please A) provide the year it was imported and B) indicate whether permits were obtained to import/export the materials used, C) provide details of any permits obtained. If this information is not available, please indicate this.

N/A

If you used archival specimens, please state how the material used in your study was acquired by the institute it is held in and provide details of any permits obtained for the original excavations/ sample collection. If this information is not available, please indicate this.

N/A

How was the potential cultural significance of the materials collected in your study to local communities considered in your research design? Were Indigenous peoples and/or local researchers and institutions involved with archaeological excavations / collection of specimens? If so, please provide a description of their involvement.

N/A

If your manuscript includes photographs of human remains please indicate whether authors obtained permission from descendants or affiliated cultural communities to do so.

N/A

**S3 Table: Evaluation of provider knowledge of pupillometry and ICP monitoring after education session (during pre-training interview) and after training session (during post-training interview)**

| Item | Number of Providers | Percent of Providers |
| --- | --- | --- |
| Described the importance of ICP monitoring for assessing the clinical state of patients with TBI and making treatment decisions | 21 | 95% |
| Stated that elevated ICP is associated with poor outcomes for patients with TBI | 20 | 91% |
| Defined the pupil light response as constriction of the pupil in response to an increase in light | 14 | 64% |
| Stated that elevated ICP can cause abnormalities in the pupil response to light when explaining relationship between pupil light response and elevated ICP | 17 | 77% |
| Named the pupillometer as the device discussed in the education session | 16 | 73% |
| Discussed association between abnormal pupillometry readings and elevated ICP | 18 | 82% |
| Described pupil size as a measurement obtained from pupillometry | 17 | 77% |
| Described NPi® as a measurement obtained from pupillometry | 9 | 41% |

**S4 Figure: Overview of themes related to perception of pupillometry
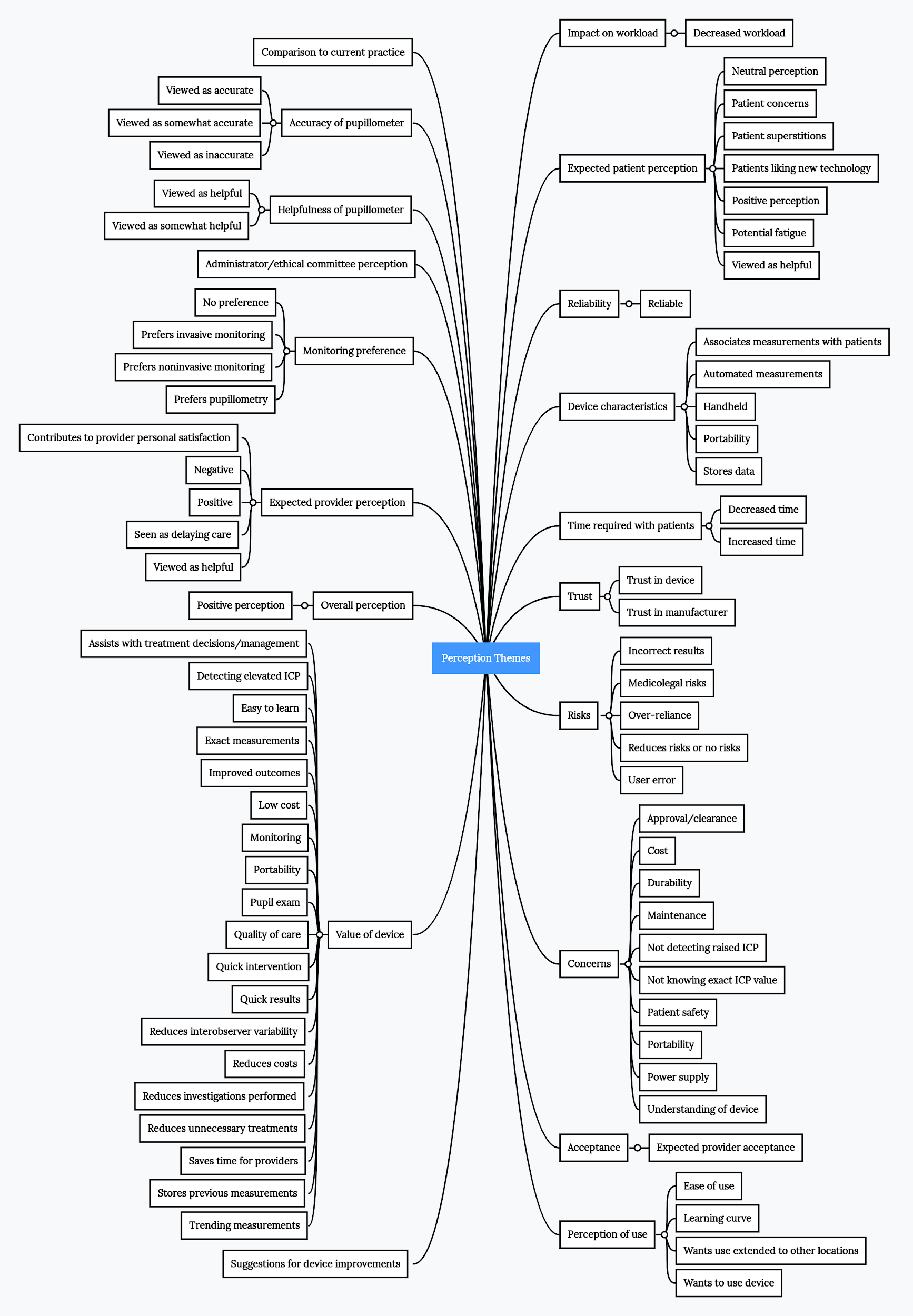
**

**S5 Figure: Overview of themes related to implementation of pupillometry
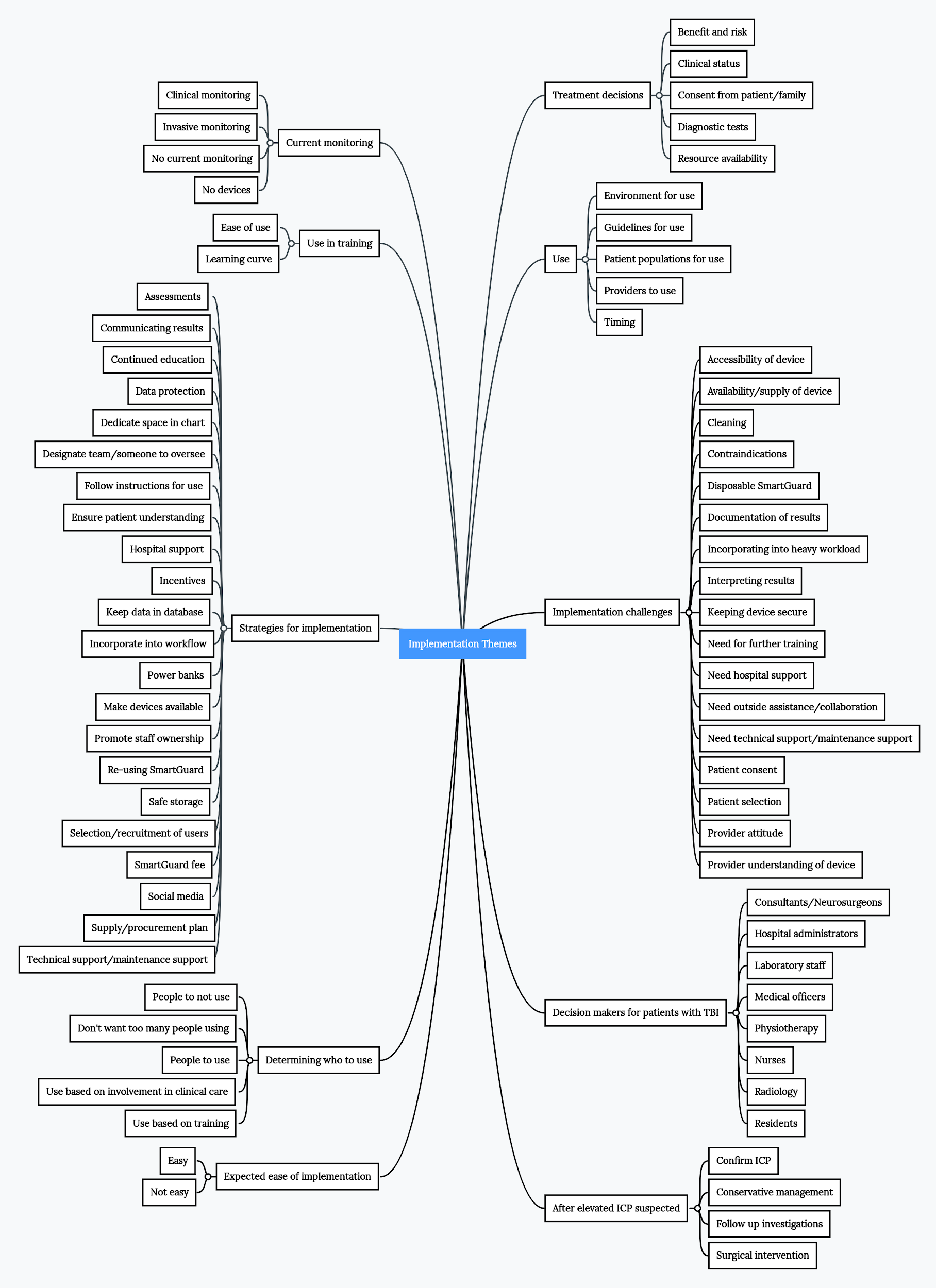
**

**S6 Table: Frequency of all perception themes identified during the qualitative interviews**

| **Parent Code** | **Primary Child Code** | **Secondary Child Code** | **Count** | **Percent** |
| --- | --- | --- | --- | --- |
| Acceptance | Expected provider acceptance | Difficult provider acceptance | 2 | 9 |
|  | Expected provider acceptance | Easy provider acceptance | 4 | 18 |
| Accuracy of pupillometer | Viewed as accurate |  | 15 | 68 |
|  | Viewed as inaccurate |  | 3 | 14 |
|  | Viewed as somewhat accurate |  | 9 | 41 |
| Administrator/ethical committee perception |  |  | 1 | 5 |
| Comparison to current practice |  |  | 14 | 64 |
| Concerns | Approval/clearance |  | 3 | 14 |
|  | Cost | Cost of device | 12 | 55 |
|  | Cost | Cost of SmartGuards | 4 | 18 |
|  | Cost |  | 1 | 5 |
|  | Durability |  | 7 | 32 |
|  | Maintenance |  | 14 | 64 |
|  | Not detecting raised ICP |  | 1 | 5 |
|  | Not knowing exact ICP value |  | 4 | 18 |
|  | Patient safety |  | 6 | 27 |
|  | Portability |  | 3 | 14 |
|  | Power supply |  | 8 | 36 |
|  | Understanding of device |  | 13 | 59 |
| Device characteristics | Associates measurements with patients |  | 2 | 9 |
|  | Automated measurements |  | 7 | 32 |
|  | Handheld |  | 6 | 27 |
|  | Portability |  | 1 | 5 |
|  | Stores data |  | 7 | 32 |
| Helpfulness of pupillometer | Viewed as helpful |  | 17 | 77 |
|  | Viewed as somewhat helpful |  | 5 | 23 |
| Impact on workload | Decreased workload |  | 9 | 41 |
| Monitoring preference | No preference |  | 5 | 23 |
|  | Prefers invasive monitoring |  | 3 | 14 |
|  | Prefers noninvasive monitoring |  | 5 | 23 |
|  | Prefers pupillometry |  | 5 | 23 |
| Overall perception | Positive perception |  | 15 | 68 |
| Expected patient perception | Neutral perception |  | 3 | 14 |
|  | Patient concerns |  | 8 | 36 |
|  | Patient superstitions |  | 1 | 5 |
|  | Patients liking new technology |  | 4 | 18 |
|  | Positive perception |  | 14 | 64 |
|  | Potential fatigue |  | 1 | 5 |
|  | Viewed as helpful |  | 1 | 5 |
| Perception of use | Ease of use | Easy to use | 13 | 59 |
|  | Ease of use | Not easy to use | 2 | 9 |
|  | Learning curve |  | 3 | 14 |
|  | Wants use extended to other locations |  | 3 | 14 |
|  | Wants to use device |  | 12 | 55 |
| Expected provider perception | Contributes to provider personal satisfaction |  | 1 | 5 |
|  | Negative |  | 6 | 27 |
|  | Positive |  | 17 | 77 |
|  | Seen as delaying care |  | 1 | 5 |
|  | Viewed as helpful |  | 4 | 18 |
| Reliability | Reliable |  | 5 | 23 |
| Risks | Incorrect results |  | 6 | 27 |
|  | Medicolegal |  | 1 | 5 |
|  | Over-reliance |  | 2 | 9 |
|  | Reduces risks or no risks |  | 6 | 27 |
|  | User error |  | 6 | 27 |
| Suggestions for device improvements |  |  | 3 | 14 |
| Time required with patients | Decreased time |  | 13 | 59 |
|  | Increased time |  | 3 | 14 |
| Trust | Trust in device | Previous use | 3 | 14 |
|  | Trust in device |  | 2 | 9 |
|  | Trust in manufacturer |  | 2 | 9 |
| Value of device | Assist with treatment decisions/management |  | 21 | 95 |
|  | Detecting elevated ICP |  | 19 | 86 |
|  | Easy to learn |  | 4 | 18 |
|  | Exact measurements |  | 2 | 9 |
|  | Improved outcomes |  | 10 | 45 |
|  | Low cost |  | 6 | 27 |
|  | Monitoring | Monitoring treatment effect | 5 | 23 |
|  | Monitoring |  | 11 | 50 |
|  | Portability |  | 2 | 9 |
|  | Pupil exam |  | 12 | 55 |
|  | Quality of care |  | 5 | 23 |
|  | Quick intervention |  | 17 | 77 |
|  | Quick results |  | 17 | 77 |
|  | Reduces interobserver variability |  | 10 | 45 |
|  | Reduces costs |  | 5 | 23 |
|  | Reduces investigations |  | 5 | 23 |
|  | Reduces unnecessary treatments |  | 5 | 23 |
|  | Saves time for providers |  | 3 | 14 |
|  | Stores previous measurements |  | 4 | 18 |
|  | Trending measurements |  | 3 | 14 |

**S7 Table: Frequency of all implementation themes identified during the qualitative interviews**

| **Parent Code** | **Primary Child Code** | **Secondary Child Code** | **Count** | **Percent** |
| --- | --- | --- | --- | --- |
| After elevated ICP suspected | Confirm ICP |  | 3 | 14 |
|  | Conservative management |  | 16 | 73 |
|  | Follow up investigations |  | 7 | 32 |
|  | Surgical intervention |  | 15 | 68 |
| Current monitoring | Clinical monitoring |  | 17 | 77 |
|  | Invasive monitoring |  | 2 | 9 |
|  | No current monitoring |  | 1 | 5 |
|  | No devices |  | 8 | 36 |
| Decision makers for patients with TBI | Consultants/Neurosurgeons |  | 18 | 82 |
|  | Hospital administrators |  | 2 | 9 |
|  | Laboratory staff |  | 1 | 5 |
|  | Medical officers |  | 3 | 14 |
|  | Nurses |  | 8 | 36 |
|  | Physiotherapy |  | 1 | 5 |
|  | Radiology |  | 1 | 5 |
|  | Residents |  | 10 | 45 |
| Determining who to use | Don't want too many people using |  | 2 | 9 |
|  | People to not use | Medical officers | 1 | 5 |
|  | People to not use | Nurses | 1 | 5 |
|  | People to not use | Nursing assistants | 1 | 5 |
|  | People to not use | Other hospital staff | 4 | 18 |
|  | People to not use | Patients/families | 2 | 9 |
|  | People to not use | Students | 2 | 9 |
|  | People to not use | Other | 1 | 5 |
|  | People to use | Doctors | 15 | 68 |
|  | People to use | Interns | 4 | 18 |
|  | People to use | Medical officers | 8 | 36 |
|  | People to use | Nurses | 12 | 55 |
|  | People to use | Others to use | 2 | 9 |
|  | People to use | Residents | 9 | 41 |
|  | People to use | Students | 2 | 9 |
|  | Use based on involvement in clinical care |  | 12 | 55 |
|  | Use based on training |  | 12 | 55 |
| Expected ease of implementation | Easy |  | 9 | 41 |
|  | Not easy |  | 2 | 9 |
| Implementation challenges | Accessibility of device |  | 5 | 23 |
|  | Availability/supply of device |  | 19 | 86 |
|  | Cleaning |  | 3 | 14 |
|  | Contraindications |  | 7 | 32 |
|  | Disposable SmartGuard® |  | 3 | 14 |
|  | Documentation of results |  | 3 | 14 |
|  | Incorporating into heavy workload |  | 3 | 14 |
|  | Interpreting results |  | 4 | 18 |
|  | Keeping device secure |  | 5 | 23 |
|  | Need for further training |  | 9 | 41 |
|  | Need hospital support |  | 1 | 5 |
|  | Need outside assistance/collaboration |  | 2 | 9 |
|  | Need technical support/maintenance support |  | 6 | 27 |
|  | Patient consent |  | 2 | 9 |
|  | Patient selection |  | 2 | 9 |
|  | Provider attitude |  | 2 | 9 |
|  | Provider understanding of device |  | 6 | 27 |
| Strategies for implementation | Assessments |  | 1 | 5 |
|  | Communicating results |  | 1 | 5 |
|  | Continued education | Manual | 3 | 14 |
|  | Continued education |  | 19 | 86 |
|  | Data protection |  | 1 | 5 |
|  | Dedicate space in chart |  | 1 | 5 |
|  | Designate team/someone to oversee |  | 7 | 32 |
|  | Ensure patient understanding |  | 6 | 27 |
|  | Follow instructions for use |  | 1 | 5 |
|  | Hospital support |  | 11 | 50 |
|  | Incentives |  | 1 | 5 |
|  | Incorporate into workflow |  | 4 | 18 |
|  | Keep data in database |  | 1 | 5 |
|  | Make devices available |  | 6 | 27 |
|  | Power banks |  | 1 | 5 |
|  | Promote staff ownership |  | 2 | 9 |
|  | Re-using SmartGuard® |  | 1 | 5 |
|  | Safe storage |  | 4 | 18 |
|  | Selection/recruitment of users |  | 4 | 18 |
|  | SmartGuard® fee |  | 1 | 5 |
|  | Social media |  | 1 | 5 |
|  | Supply/procurement plan |  | 2 | 9 |
|  | Technical support/ maintenance support |  | 7 | 32 |
| Treatment decisions | Benefit and risk |  | 2 | 9 |
|  | Clinical status |  | 11 | 50 |
|  | Consent from patient/family |  | 1 | 5 |
|  | Diagnostic tests | Imaging | 8 | 36 |
|  | Diagnostic tests | Labs | 1 | 5 |
|  | Diagnostic tests |  | 2 | 9 |
|  | Resource availability |  | 4 | 18 |
| Use | Environment for use |  | 6 | 27 |
|  | Guidelines for use |  | 5 | 23 |
|  | Patient populations for use | Caution | 7 | 32 |
|  | Patient populations for use | Ideal patient | 21 | 95 |
|  | Patient populations for use | Outside of TBI | 10 | 45 |
|  | Patient populations for use | Patients to avoid | 11 | 50 |
|  | Providers to use | Can be used by anyone | 4 | 18 |
|  | Timing | Regular repetition | 17 | 77 |
|  | Timing | Time of day | 3 | 14 |
|  | Timing | Timing based on patient condition | 14 | 64 |
|  | Timing | Upon presentation | 8 | 36 |
|  | Timing |  | 2 | 9 |
| Use in training | Ease of use | Easy to use | 5 | 23 |
|  | Ease of use | Not easy to use | 1 | 5 |
|  | Learning curve |  | 5 | 23 |

**S8 Table: Exemplary quotes related to perception themes and role of provider***

| Parent Code | Primary Child Code | Secondary Child Code | Quote | Role |
| --- | --- | --- | --- | --- |
| Accuracy of pupillometer | Viewed as accurate |  | First of all, it [pupillometry] is going to give us accurate findings for pupillary exam. | Resident |
| Comparison to current practice |  |  | I think it's a good device to use and why I think so is because it's, it's a device that's employs what you've been doing before only that it is making it more meaningful by putting figures and meaning to it. Because usually, it will be a subjective assessment. | Resident |
| Concerns | Cost | Cost of device | Others don’t like to attach a cost to patient care, so that may also be a barrier, so they would prefer using their flashlights because it doesn’t cost anything. The device costs some money, and you have to buy a memory card every time you use it and it has to be maintained, so they would prefer to use a cheaper way of doing it. | Neurosurgeon |
| Concerns | Maintenance |  | Then another thing is the challenge of its maintenance. We, we don't know how. We haven't used it. […] We don’t know the risks and how to repair it and how to maintain it to make, to make a sustainable way of monitoring intracranial pressure in TBI. | Resident |
| Concerns | Understanding of device |  | My only concern is the knowledge of the device. | Resident |
| Helpfulness of pupillometer | Viewed as helpful |  | I think they are very helpful, because they guide you to monitor your ICP because that was your intention. So if you got a better way of examining a patient you get a guidance, way forward, monitoring the patient. | Nurse |
| Overall perception | Positive perception |  | My overall perception, I perceive that it is going to, ease my work, improve the patient care and outcome by giving us quick intervention, accurate monitoring and therefore it is, it will be dependable. If it gives us an accurate intervention, we will depend a lot on that machine. Yes. And maybe Mulago, having piloted it if it is good, we would wish to see our other colleagues in other areas extend the what? The use of the device, since they refer to us, so that we are at the same level in the management of the patient, so that we speak that same language. | Nurse |
| Expected patient perception | Positive perception |  | They will like it a lot because most of our patients like to discover, like to understand what is being done there and they believe is always if you bring a device to work on them then you are doing something good to make them improve as fast as possible. I think they will like it. And also, the fact that it is being noninvasive, you're not causing any harm to them. | Nurse |
| Perception of use | Ease of use | Easy to use | It was easy if you mastered it, if you placed it properly, it’s easy. That is in patients that are not fighting or not restless, confused, I think it's not bad, provided you must have a way to use it. It's not difficult. | Nurse |
| Perception of use | Wants to use device |  | You are doing a feasibility study. I hope that after this discussion, personally, I can get that tool because for my personal clinical assessment of my patient, I think that it can make a difference, can make a difference when I'm reviewing patient, out, even neurosurgical out clinic. You know, we have many patients, you need to be sure of when you are dealing with any patient with a space occupying lesion, you need to be sure what is going on. I think that's one can save time and also make the decision more objective than just that clinical assessment which is obvious, you know. But in terms of the measurements, that's, while, that points specifically, you have difference among assessors. Yes, because myself I think that is a good tool. We need to have it now in our daily clinic. It can make difference, yes. | Resident |
| Expected provider perception | Positive |  | But overall I think most of my colleagues will find it acceptable. Because it's better than what is, what we normally do before the CT scan is done. Look at pupils at times there, times, uh, times you're not able to assess pupils. Sometimes you see the pupils and we think, oh, there is no ICP, then it's there. | Neurosurgeon |
| Time required with patients | Decreased time |  | The benefits will include, first of all, early detection of raising intracranial pressure compared to, to our use of the pen torch and clinical, just clinical, our clinical examination findings. Yes. | Resident |
| Value of device | Assist with treatment decisions/management |  | It can be used for monitoring. The values that you got, you match it to the new values that the patient has then you see the trend that there is actually increase in ICP even before you get to a critical point. So I think it will help us make decisions quicker. | Resident |
| Value of device | Detecting elevated ICP |  | The benefits will include, first of all, early detection of raising intracranial pressure compared to, to our use of the pen torch and clinical, just clinical, our clinical examination findings. | Resident |
| Value of device | Monitoring |  | It can be used for monitoring. The values that you got, you match it to the new values that the patient has then you see the trend that there is actually increase in ICP even before you get to a critical point. | Resident |
| Value of device | Pupil exam |  | The device monitors the pupil reflex time. How long is the reflex take for that particular patient? To constrict maximally and also come back to normal pupil aperture | Laboratory technician |
| Value of device | Quick intervention |  | I just need to choose a quick intervention, quick intervention. And then it will lead to good outcomes. It will lead to good outcomes because there will be quicker, quick, timely intervention. | Resident |
| Value of device | Quick results |  | I think they can benefit a lot because the device helps us to quickly identify patients, we suspect might have suffered traumatic brain injury and who we think have raised intracranial pressure. Within the matter of minutes we have taken a measurement and it’s able to tell us whether our patient has raised intracranial pressure or not. So, I think we can benefit a lot from the device. | Nurse |

*Only themes that were discussed by 11 or more participants (>50%) were included. Refer to S6 Table for additional themes.

**S9 Table: Exemplary quotes related to implementation themes and role of provider***

| Parent Code | Primary Child Code | Secondary Child Code | Quote | Role |
| --- | --- | --- | --- | --- |
| After elevated ICP suspected | Conservative management |  | We always give medications that reduce ICP. And majorly we use mannitol and hypertonic saline. And of course restrict those that increase the pressure, like giving the dextrose 5%. We restrict them. | Nurse |
| After elevated ICP suspected | Surgical intervention |  | [...] the surgical management is also another option because if the patient has space occupying lesion, you know, we need to deal with that lesion for us to expect to reduce ICP for that particular patient. | Resident |
| Current monitoring | Clinical monitoring |  | Currently we depend on the blood pressure of the patient and history taking. And we see how has been this patient before, how is the patient now. If the blood pressure is high. So in case of high blood pressure we suspect there is increased intracranial pressure. Then with the pupils. Yeah. Majorly those are the things we use. | Nurse |
| Determining who to use | People to use | Doctors | I think any, anyone involved in patient day to day care. That includes the doctors, the nurses, what you’d call physician assistants. I think they should all be involved. | Neurosurgeon |
| Determining who to use | People to use | Nurses | You will use it, many will use it because as long as they are trained, most especially the critical care nurses. | Nurse |
| Determining who to use | Use based on involvement in clinical care |  | Those are the best people [to use the device] because they are the people who are in direct contact with these patients, involved in their care. | Resident |
| Determining who to use | Use based on training |  | That’s the first thing and then also people who are using it basically people are trained. If you don't know how the device is operated, it might be challenging. So, I feel people have the knowledge on how to use the device should be the one to use it. | Nurse |
| Implementation challenges | Availability/ supply of device |  | And we are not sure, after the study if the equipment, pupillometries, are going to stay for our use in the department. So all that we are unsure. So we are waiting for the implementation process to see what should happen, whether we should stay with our own after implementation. Or they will procure for us more. Or the administration will buy the idea after seeing what the results are and then they procure for themselves. And how to maintain to have a sustainable way of what? Monitoring ICP for our TBI patients. | Resident |
| Strategies for implementation | Continued education |  | The training for the device means making sure the training is continuous, either within the group that has been trained, we can organize CMEs for the next groups. I think these are some of the ways. | Resident |
| Strategies for implementation | Hospital support |  | Making the administration and hospital boards buy the idea, accept its use such that it is not, it is made legal. And then another thing, is by the management, by the management accepting it, it might be made compulsory as a routine assessment for every TBI patient, such that it is in the system. So that for every patient you have to first get to know the NPI, NPI values for that patient. So the institution can push it with proper training. | Resident |
| Use | Patient populations for use | Ideal patient | And also, we need to use the device as we are doing, we review our patients, and the patient in ICU, you may need that [pupillometry] device to be used. Patients in our general ward, because we can receive your patients with small hematoma admitted in the ward but the situation may change. As we are reviewing the patients regularly. We may need to have objective tool with us. And I think is a continuation use. | Resident |
| Use | Patient populations for use | Patients to avoid | I don't know if it would make sense in people who have had orbital trauma, and then the patients who we are thinking are brain dead. | Neurosurgeon |
| Use | Timing | Regular repetition | Any patient who has TBI, I would use the device on the initial assessment, but then a serial assessment, I'll use it on patients whom I asked I suspect have increased the ICP. | Resident |
| Use | Timing | Timing based on patient condition | Even the monitor, the frequency of monitor differs patient by patient. We need also to consider now those normal patients just we are reviewing patient after the acute phase of the of injury. You know, those patients now after the acute phase, they are different. That mean the answer is defer, according to, which category of patients, we are dealing with. | Resident |

*Only themes that were discussed by 11 or more participants (>50%) were included. Refer to S7 Table for additional themes.

**S10 Table: Full data on learning time and accuracy measurements from the pupillometry training session**

| Learning Time (seconds) | Participant NPi Measured in Left Eye | Trainer NPi Measured in Left Eye | Participant NPi Measured in Right Eye | Trainer NPi Measured in Right Eye | Participant Size Measured in Left Eye | Trainer Size Measured in Left Eye | Participant Size Measured in Right Eye | Trainer Size Measured in Right Eye |
| --- | --- | --- | --- | --- | --- | --- | --- | --- |
| 749.6 | 3.9 | 3.9 | 3.6 | 3.7 | 3.36 | 3.52 | 4.08 | 3.6 |
| 600.1 | 3.9 | 3.9 | 4.2 | 4 | 3.34 | 3.34 | 3.23 | 3.29 |
| 769 | 4.1 | 4.1 | 3.8 | 4.1 | 3.06 | 3 | 2.95 | 3.05 |
| 704.1 | 3.7 | 3.8 | 3.8 | 4.1 | 2.98 | 2.57 | 2.57 | 2.82 |
| 812.1 | 4.2 | 4.3 | 4.5 | 4 | 4.28 | 4 | 3.69 | 3.56 |
| 764.7 | 4.3 | 4.2 | 4.4 | 4.4 | 4.64 | 4.28 | 3.61 | 3.63 |
| 694.6 | 4 | 4.1 | 4.1 | 4.2 | 4.76 | 4.43 | 4.56 | 4.37 |
| 613 | 4 | 3.9 | 3.9 | 4.2 | 5.51 | 4.65 | 5.15 | 5 |
| 846.9 | 4.4 | 4.2 | 4.3 | 4.4 | 3.07 | 4.85 | 3.44 | 3.16 |
| 1379.3 | 4.5 | 4.5 | 4.5 | 4.4 | 2.86 | 2.81 | 3.04 | 2.75 |
| 922.2 | 4.8 | 4.7 | 4.7 | 4.6 | 2.97 | 3.12 | 3.52 | 3.34 |
| 883 | 4.6 | 4.7 | 4.7 | 4.6 | 3.26 | 3.12 | 3.51 | 3.69 |
| 675 | 4.6 | 4.6 | 4.6 | 4.5 | 2.5 | 2.74 | 2.67 | 2.7 |
| 762.1 | 4.1 | 4.3 | 4.4 | 4.2 | 2.63 | 2.78 | 2.94 | 2.72 |
| 1146 | 4.7 | 4.7 | 4.6 | 4.7 | 3.54 | 3.1 | 2.95 | 3.27 |
| 582.1 | 4.5 | 4.6 | 4.5 | 4.2 | 2.57 | 2.64 | 2.97 | 2.87 |
| 582.1 | 4.1 | 4 | 3.8 | 4 | 3.03 | 3.15 | 3.36 | 3.8 |
| 1068.5 | 4.5 | 4.6 | 4.5 | 4.4 | 2.33 | 2.34 | 2.68 | 2.66 |
| 998.5 | 3.6 | 3.2 | 3.4 | 3.4 | 3.26 | 3.39 | 3.4 | 3.11 |
| 735.9 | 4.4 | 4.3 | 4.3 | 4.3 | 3.27 | 3.46 | 3.47 | 3.41 |
| 713.2 | 4.6 | 4.4 | 4.5 | 4.6 | 2.69 | 2.78 | 2.47 | 2.7 |
